# The countercyclical long-term operating accrual-based trading strategy in the Stoxx Europe 600 index: The importance of asset and liability components

**DOI:** 10.1371/journal.pone.0266045

**Published:** 2022-05-26

**Authors:** Alberto Sandoval, Javier Márquez, Ignacio Cervera

**Affiliations:** 1 Department of Finance, United International Business Schools, Madrid, Spain; 2 Department of Finance, Universidad Pontificia Comillas, Madrid, Spain; The Bucharest University of Economic Studies, ROMANIA

## Abstract

This work uses long-term operating accruals, rather than current, as an accounting measure to identify major anomalies. Past and abundant accounting and financial literature associates anomalies with problems of reliability and assigns lower reliability to long-term operating accruals than to current accruals. We investigate the relation between scaled operating accruals and size-adjusted abnormal returns for nonfinancial firms listed in the Stoxx Europe 600 index for the period 2000–2021. We find consistent evidence of (1) a higher long-term operating accrual anomaly than working capital accrual, especially, when asset and liability components are separated (2) long-short trading strategies aimed at taking advantage of the anomaly that achieves significant annual returns between 2% and 6% and (3) this trading strategy strongly reduces the risk of stock portfolios during an economic crisis due to its countercyclical nature. These findings have important implications not only for academics, but also for asset managers who want to protect the return of their stock portfolios from high market volatility.

## 1. Introduction

To measure their firm’s current-period operating performance, accountants compute earnings by adding adjustments called accruals to free cash flow [1]. Sloan [2] first documented a negative relationship between current accruals and stock returns in the USA, identifying an accrual anomaly due to incorrect investor valuations based on the accounting information contained in those accruals. Since then, Larson, *et al*. [3] documented more than a hundred articles in leading accounting journals with the word ‘accrual’ in their title, with many classifications of accruals for different purposes. In December 2020, we found 378 articles in the Web of Science in Business and Finance with the word ‘accrual’ in their abstract. Most of these articles aim to determine how and why these accruals negatively predict the cross-section of stock returns.

Traditional literature on the accrual anomaly focuses on current and total accruals, especially in US markets. Very little has been documented on the long-term accrual anomaly in Europe. To the best of our knowledge, no specific behavioral tests have been carried out of the noncurrent assets and liabilities accrual anomalies during economic cycles, which is the focus of this article. According to Sloan [2], a lower reliability of accruals increases the anomaly. Richardson *et al*. [4] and Downes *et al*., [5], provide evidence that long-term accruals are subject to a greater degree of subjectivity and hence are less reliable than current accruals, while Zhu [6] stated that operating assets are the least reliable accrual components. Chichernea *et al*. [7] observed that long-term components of accruals are the most robust predictor of returns, especially their asset constituent.

Relatively little work has been done on the accrual anomaly outside the USA. Iñiguez & Poveda [8] find a higher persistence difference between cash flows and accruals in Anglo-Saxon accounting systems, possibly related to the stronger implementation of the fair value criteria, and observe higher accrual anomalies than in European accounting systems. Following this vein, we expect to find lower abnormal returns in nonfinancial firms of the Stoxx Europe 600 Index than those found in other Anglo-Saxon studies.

Chichernea *et al*. [7] suggest that a bond accrual strategy is time-varying, and countercyclical and can be predicted by return dispersion. They find that stock return dispersion increases prior to and during periods of recession, and correlates positively with bond hedge returns. This correlation is higher for noncurrent than for working capital accruals. Consistent with this idea, Gonçalves *et al*. [9] find that the accrual anomaly shows asymmetric differential persistence for accruals and cash flows in years of economic losses and economic gains. Zhu [6] found that high accruals predict a higher probability of future price crashes than low accruals.

We investigate whether this countercyclical behavior observed in the USA in bond hedge returns extends in Europe to stock hedge returns and if so, what differences are observable between noncurrent and working capital accruals.

Our goal is to investigate the possible extension of the anomaly to long-term operating accruals in European firms, and specifically to their constituents’ long-term operating asset and liability accruals during recession and revival periods. We theorize that investors’ sensitivity to long-term operating asset accruals is higher than to long-term operating liability accruals because the former is more related to recurring profits than the latter. Hence, the separation of long-term accruals into their asset and liability components may yield better estimates of abnormal returns. Following Zhu [6], we also theorize that high operating asset accrual is a better predictor of price crashes, so it can be used in counter cycle-based strategies to minimize volatility and risk.

To achieve that goal, we first calculate the abnormal returns of each stock of the Stoxx Europe 600 ex Financial sector, from 2001 to 2021, to the returns of five portfolios built by market size (quintiles). Second, we distribute all stocks into four new portfolios (quartiles) sorted by accruals (working capital accruals, noncurrent accruals and their components, assets and liabilities accruals) from lower to higher, and scaled by total assets. Third, we compare which accruals are followed by higher abnormal returns, using a conventional statistical approach and running linear regression analysis. Last, we observe if an accrual-based trading strategy, consistent with buying low and selling high accrual stocks, is capable of delivering superior hedge returns compared with the Stoxx Europe 600 performance during the last 21 years, especially during crisis periods, where the proposed strategy performs as a countercyclical strategy.

Our empirical findings contribute to the current literature in several important ways. The foremost is our focus on long-term operating accruals in European listed companies and the information contained in their asset and liability components as better variables to detect anomalies. We find important volatility reductions in stock portfolio hedge returns that follow a long-term operating asset accrual trading strategy, in line with Chichernea *et al*. [7] for USA bonds. To the extent of our knowledge, this is the first paper to include this novel approach with great potential for academics and practitioners.

This work is divided into five sections. The second section reviews the scientific literature related to the subject and discusses the limitations that inspire this research. The third section describes the methodology used. The fourth section explains the empirical results and discussion and the last section concludes.

## 2. Literature review and hypothesis development

### 2.1. Accruals

Accruals represent the essential elements supporting the activity of the firm [10] and are the result of management estimates of the value of corporate assets and liabilities in an accounting system based on the accrual principle. Because they are part of the company’s profit, estimation errors directly affect the determination of profit. To mitigate these potential problems, Jones [11] developed a model that indirectly measures operating accruals based on sales and long-term assets, and promoted an investment philosophy based on cash flows rather than profits.

Accruals contain important information for the future earnings of the firm, so an observer who misinterprets these adjustments will have a distorted view of the firm´s future prospects and may overvalue firms with high accruals or undervalue those with low accruals [12].

### 2.2. The accrual anomaly

Keim [13] defines anomalies in financial markets as patterns seen in a series of returns that differ from those predicted by a major theory. The abundant literature interprets the accrual anomaly around two opposing main hypotheses that offer the same prediction for abnormal returns but different forecasts for future profits: the persistence hypothesis [2, 4, 14], which indicates that accruals are negatively associated with future profits because they are less persistent than cash flows; and the growth hypothesis that predicts, under optimal investment, a positive relationship between accruals and profit growth [10]. Detzel *et al*. [1] distinguish two accrual anomalies: a risk-based premium for accruals that capture real investment–related to the growth hypothesis -, and short-lived mispricing of accruals that capture transitory adjustments to profitability—related to the persistence hypothesis.

Alternative theories explain the anomaly due to agency costs and earnings management manipulation or value/growth and external financing anomalies.

The theory of agency costs of overvalued equity [15] adds that high accruals are, in part, a consequence of past pricing errors that [16] motivate upward management manipulations of accruals that will undermine, when reverted, future profits and returns. None of this occurs in low accruals. Accruals are thus subject to earnings management or even fraud [13, 17, 18] because it is much easier for management to distort profits than cash flows. Aldahray & Alnori [19] found that managers manipulate accruals in bankrupt firms, especially in lightly regulated markets compared to more regulated ones. Li *et al*. [20] observe that highly valued Chinese state-owned enterprises show higher abnormal accruals than highly valued non-state-owned enterprises.

The accrual anomaly is related to the value/growth anomaly and the external funding anomaly. According to the value/growth anomaly, investments in undervalued companies generally offer higher future returns than those in growth companies. The external funding anomaly predicts that shares of companies that receive external funding are usually less profitable than shares of those who distribute it. Some researchers [21, 22] claim that the value anomaly is the fundamental cause of the external funding anomaly and that the accrual anomaly is a consequence of the first two.

The importance of the accrual anomaly derives mainly from its characteristics that award high potential financial advantages to trading strategies based on the accrual anomaly. The first is its economic relevance, with hedge returns above 10% in studies carried out in the USA [2, 23, 24]. Second, despite being widely studied and known, its persistence over time is attributed to the unattractive characteristics of the firms that yield the highest hedge returns [25]. Third, the perceived independence of hedge returns obtained with accrual-based trading strategies from general stock market returns, and fourth, its global reach. However, various international studies mainly confine its presence to Anglo-Saxon countries [26–28], or the UK [29] while some studies [8, 30] do not detect abnormal returns in certain countries or detect them in an inconclusive or weak form. These international differences may be explained by the variation in sales growth persistence, accounting practices, and sample size [31].

### 2.3. Hypothesis

Past studies of the anomaly mainly concentrated on current accruals, those related to assets and liabilities with a less than one-year maturity. Very few studies have focused on the European Market between 2000 and 2021, when periods of economic recession and revival coexist. This paper proposes a different approach by focusing not only on long-term operating accruals, but also on the information supplied by its noncurrent asset and noncurrent liability components (those related to a more than one-year maturity) in European firms during this period, and whether such information may take advantage in counter cycling trading strategies.

Since Sloan [2], most of the accrual literature has focused on working capital accruals. Nevertheless, working capital cannot capture accounting distortions associated with long-term accruals [18]. If lower reliability of accruals increases the anomaly [2] and long-term accruals are subject to a greater degree of subjectivity (as firms must decide what type of expenses should be capitalized, R&D, goodwill impairment loss, write-downs, and, for long-term liabilities, estimate pension benefit obligations and other fair value estimates for assets or liabilities), and are hence less reliable than current accruals [4, 5], then long-term accruals have the potential to identify greater abnormal returns than working capital accruals.

Then, our first hypothesis is as follows:

H1: The long-term operating accrual anomaly is higher than the working capital accrual anomaly.

Current literature documents an accrual anomaly related to investments in long-term assets but pays little attention to long-term operating liabilities. There is a positive correlation of profits with investments [32], and companies that substantially increase their capital investments and asset growth obtain negative adjusted returns [33–35]. However, there is a negative correlation with external finance, with weak correlations between investing and financing activities [32]. Nevertheless, external financing accruals are not part of long-term operating liability accruals.

Since the anomaly is a consequence of investor mispricing errors, those who put a different focus on long-term operating assets and liability accruals may react distinctively to future corrections of their respective values and their consequential impact on profits. We theorize that investors’ sensitivity to long-term liability accruals is lower than to long-term operating asset accruals. Long-term liabilities´ relatively smaller size (mainly composed of provisions, warranties, deferred taxes, and minority interests) and their lower relationship to recurring earnings may decrease investors´ interest and reactions when they prove incorrect by future write-downs or write-ups that affect future profits. On the other hand, long-term operating assets, mainly composed of intangible assets, property, plants and equipment, are more intensely related to the firm´s ordinary activities as necessary investments to conduct operations and to eventually increase profits. Their failure to do so significantly compromises future recurring earnings and may drive sharper adverse investor reactions.

Then, our second hypothesis is as follows:

H2: The decomposed long-term operating asset accrual anomaly is higher than the long-term operating liability accrual anomaly.

Recent literature studies the relationship of the accrual anomaly to market volatility and its countercyclical behavior. A bond accrual strategy is time-varying, countercyclical, and can be predicted by return dispersion, which increases prior to and during periods of recession and correlates positively with bond hedge returns [7, 9]. During the European sovereign debt crisis, the accrual anomaly appeared to exist only in periods of economic gains [9] and it did not appear in revival economic cycles. Moreover, we can obtain positive accrual premia in medium or high sentiment times for investment accruals and in low sentiment times for working capital and nontransaction accruals [1]. More specifically, higher operating asset accruals predict a higher price crash probability than low accruals [6].

According to these recent studies, we theorize that an accrual-based trading strategy could deliver steadier returns than a sample trading strategy, because the latter would be fully subject to stock market volatility. An accrual-based trading strategy forms annual buy-and-hold portfolios of long positions in low accrual stocks and short positions in high accrual stocks. A sample trading strategy creates annual buy-and-hold portfolios of long positions of all sample stocks.

Our third hypothesis is as follows:

H3: An accrual-based trading strategy generates lower volatility of stock returns than a sample trading strategy, and it can be used as a countercyclical-based trading strategy.

## 3. Materials and methods

This work evaluates the presence of the accrual anomaly in the nonfinancial firms listed in the Stoxx Europe 600 index. We compare working capital accruals with noncurrent (long-term) accruals, decomposing this into its assets and liabilities components.

To control for the well documented negative size effect on stock returns [36–38], this study first calculates the abnormal returns of each referenced stock to the returns of five portfolios built by market size (quintiles), following procedures of previous authors [2, 24, 39].

Second, it distributes all stocks into four new portfolios (quartiles) sorted by accruals from lower to higher and calculates the abnormal returns corresponding to each portfolio each year. Significance levels are analyzed using a conventional statistical approach [2], and running linear regression analysis. All accruals are calculated annually from company annual reports and scaled by total assets. We compare which type of accruals (working capital, long term assets, and long term liabilities accruals) are followed by higher abnormal returns.

Third, we form two portfolios, one composed of high and another one of low accrual stocks, and observe if an accrual-based trading strategy, consistent with buying low and selling high accrual stocks, is capable of delivering superior hedge returns compared with the Stoxx Europe 600 performance during the 21 years.

### 3.1. Sample

The accounting and financial variables were extracted from the Stoxx Europe 600 index according to data provided by Refinitiv Eikon Datastream. Stoxx Europe 600 represents large, medium and small capitalization companies from nineteen countries in the European region: Austria, Belgium, Czech Republic, Denmark, Finland, France, Germany, Greece, Ireland, Italy, Luxembourg, the Netherlands, Norway, Poland, Portugal, Spain, Sweden, Switzerland, and the UK. The number of Stoxx Europe 600 index components is fixed at 600.

To simulate an implementable trading strategy, this research uses a portfolio formed on the 2nd of May of each year, based on prior-year accounting information and excluding those stocks belonging to the financial sector and those without complete annual information available on the database. It is not necessary that stocks belong to the index during the whole year before the event, only that there is sufficient data in the database to perform the required calculations in each portfolio formation event.

The study period extends from December 31, 1999, to December 31, 2019. As the calculation of stock returns begins four months after the year-end, the annual return computations start between May 2, 2000, and May 2, 2020, and the last quote is observed in May 2021. From a total of 12,600 stock-year observations (600 x 21 years), the final sample is composed of 9,239 observations, after removing the financial sector and those with missing accounting data, which means an average of 440 companies per year.

### 3.2. Variables

#### 3.2.1 Accruals

Richardson *et al*. [4] defined total accruals (TACC) as the change in net working capital (ΔWC) plus the change in net noncurrent operating assets (ΔNCO) plus the change in net financial assets (ΔFIN):

TACC=ΔWC+ΔNCO+ΔFIN (1)

This work uses two main accrual factors:

1. Working capital accruals (WCACC).
2. Noncurrent (long-term) operating accruals (LTOACC), also decomposed into their constituent noncurrent operating asset accruals (NCOAACC) and noncurrent operating liabilities accruals (NCOLACC).

We use the definition of working capital accruals by Larson *et al*. [3]:

WCACC=ΔWC=(ΔCA−ΔCHE)−(ΔCL−ΔDLC) (2)

where WCACC are working capital accruals, ΔCA is the change in current assets, ΔCHE is the change in cash and cash equivalents, ΔCL is the change in current liabilities and ΔDLC is the change in debt in current liabilities.

Richardson *et al*. [4] computed ΔNCO as the change in noncurrent operating assets, net of long-term non-equity investments and advances (ΔNCOA), less the change in noncurrent operating liabilities, net of long-term debt (ΔNCOL):

LTOACC=ΔNCO=ΔNCOA−ΔNCOL (3)

Being:

NCOAACC)=ΔNCOA=ΔTA−ΔCA−ΔOI−ΔIIUS (4)

where ΔTA is the change in total assets, ΔCA is the change in current assets, ΔOI is the change in other investments and ΔIIUS is the change in investments in unconsolidated subsidiaries.

NCOLACC=ΔNCOL=ΔTL−ΔCL−ΔLTD (5)

where ΔTL is the change in total liabilities and shareholder equity, ΔCL is the change in current liabilities and ΔLTD is the change in long-term debt.

Accruals are scaled by average total assets. This work uses total net earnings to calculate accruals, to avoid problems of management subjectivity when classifying earnings as recurring or nonrecurring [24, 40–42]. It excludes minority interests and uses net income attributable to shareholders after extraordinary as a final measure.

#### 3.2.2 Stock returns

Returns are recorded for each stock monthly from the date of portfolio formation, which is the first business day in May of each year. Four months after the fiscal year end, almost all firms have their annual financial statements available to the public [43]. Therefore, starting in May of 2000, total returns of each stock are calculated in euros using adjusted prices with net dividends (after tax) reinvested.

### 3.3. Procedures and statistics

#### 3.3.1 Annual stock size-adjusted abnormal return calculations

Every year, market size and annual returns with net dividends included are calculated for each stock. The sample is divided into five reference portfolios of similar market size (quintiles), and the returns of all stocks in each quintile are averaged [39]. The abnormal size-adjusted return of each stock will be the difference between its return and that of its corresponding quintile. Given the large number of small firms in the sample, this division concentrates 65% of firms in the smallest portfolio. This situation is resolved by further subdividing this portfolio into five equal-size portfolios, creating nine size-reference portfolios.

The annual return of each stock is obtained, compounding its monthly returns during the twelve months following the stock portfolio formation by market size in the month of May. The annual return of each size-adjusted reference portfolio is calculated averaging the annual returns of all stocks included in the portfolio, and the annual size-adjusted abnormal return of every stock will be the difference between its annual return and the annual return of the size portfolio to which it belongs.

Note that by construction, the sum of all size-adjusted abnormal returns of each portfolio will always be zero.

#### 3.3.2 Quartile abnormal stock returns

Stocks are divided into quartiles, assigning the lowest accruals to the first quartile and the highest accruals to the last quartile. To simulate an implementable trading strategy, the breakpoints between quartiles made on the first year are respected throughout the entire period of study, so the number of stocks included in each quartile varies from year to year [24].

The annual size-adjusted abnormal return of each quartile will be the arithmetic average of the size-adjusted abnormal returns of all stocks included in the quartile. As the sample period is 21 years, the size-adjusted abnormal return in each quartile will be the arithmetic mean of the 21 annual size-adjusted abnormal returns.

#### 3.3.3 Hedge-return calculations

Hedge returns are defined as returns from an accrual-based trading strategy that consists of a buy-and-hold trading strategy in a zero-investment hedge portfolio composed of a sample stock long position in the first quartile (the low accrual portfolio) financed with a short position from the last quartile (the high accrual portfolio) held for a period of twelve months starting on May 2nd. At the end of the year, this hedge portfolio is unwound to form a new portfolio based on the new sample next year´s value of accruals. This process is repeated every year in the study period.

For the period, the size-adjusted excess hedge return will be the average of the size-adjusted annual excess hedge returns of the first quartile minus the average of the fourth quartile’s size-adjusted annual excess hedge returns.

#### 3.3.4 Test statistics

To evaluate the statistical significance of the average samples of size-adjusted abnormal returns in each quartile, p values are calculated in each quartile based on the standard deviation of the time series of the annual portfolio abnormal stock returns using a two-tailed conventional t-statistic, under the null hypothesis of no abnormal returns [2]. The significance of the sample mean of hedge returns is similarly tested by applying the t-statistic to the distribution of the 21 annual returns of the first quartile minus the 21 annual returns of the last quartile, under the null hypothesis of no hedge returns.

To complete the analysis about the presence of anomalies in certain types of accruals, and for robustness, two regression models have been carried out, as follows:

$$\text{Abn}.\;\text{Ret}._{\left(\text{t}+1\right)}=\alpha +\beta_{1}\;{\text{TWACC}}_{\text{t}}+\beta_{2}\;{\text{TLTACC}}_{\text{t}}+\text{u}$$ (6)

$$\text{Abn}.\;\text{Ret}._{\left(\text{t}+1\right)}=\alpha +\beta_{1}\;{\text{TWACC}}_{\text{t}}+\beta_{2}\;{\text{TNCOAACC}}_{\text{t}}+\beta_{3}{\text{TNCOLACC}}_{\text{t}}+\text{u}$$ (7)

where TWACC_t_ is the working capital accruals scaled by total assets in year t; TLTACC_t_ is the long-term (noncurrent) operating accruals scaled by total assets in year t; TNCOAACC_t_. is the noncurrent operating assets accruals scaled by total assets in year t; TNCOLACC_t_ in the noncurrent operating liabilities accruals scaled by total assets in year t; β_i_ is the coefficient of each type of accrual; Abn. Ret._(t+1)_ refers to the size-adjusted abnormal return of the stocks during the twelve months following the stock portfolio formation by market size in the month of May; and u is the error.

To evaluate whether the accrual-based trading strategy performs as countercyclical, especially during crisis periods, we compare the standard deviation of its performance with that of the Stoxx Europe 600 total return (including dividends) during the 21 years analyzed.

## 4. Results and discussion

Table 1 contains descriptive statistics of the sample. It includes 9,239 observations from the years 1999–2021 (1999–2019 for accounting variables; 2000–2021 for financial).

**Table 1:** Descriptive statistics.

	Average	Median	Standard Deviation	First Quartile	Third Quartile	Maximum Value
Market Capitalization (millions of euros)	13,120	5,555	22,963	2,796	12,506	305,149
Total Return (a)	11.9%	10.3%	38.5%	-11.2%	32.1%	464.7%
Abnormal Return (b)	0.0%	-1.3%	29.8%	-17.4%	15.5%	411.0%
Long term accruals (c)	0.0510	0.0244	0.1642	-0.0128	0.0773	1.8164
Working capital accruals (d)	0.0012	0.0008	0.0559	-0.0181	0.0195	0.7253
Long term operating asset accruals (e)	0.0570	0.0262	0.1776	-0.0102	0.0826	1.9212
Long term operating liability accruals (f)	0.0060	0.0027	0.0493	-0.0074	0.0157	1.2131
Stoxx Europe 600 annual returns (g)	6.25%	10.08%	20.56%	-10.49%	20.34%	36.03%

^(a)^ Total Returns of each stock are calculated in euros using adjusted prices with net dividends (after tax) reinvested. Here we include the increase in % from May 2 of fiscal year n+1 to May 2 of fiscal year n+2

^(b)^ Abnormal size-adjusted return of each stock will be the difference between its return and the average of its corresponding quintile

^(c)^ Long term operating accruals are calculated as the change in noncurrent operating assets minus the change in noncurrent operating liabilities divided by average annual total assets.

^(d)^ Working capital accruals are calculated as the change in non-cash current assets minus the change in current liabilities less short term financial debt, divided by average annual total assets.

^(e)^ Long term operating asset accruals are calculated as the change in intangible assets, property, plant, equipment and other noncurrent assets divided by average annual total assets.

^(f)^ Long term operating liability accruals are calculated as the change in minority interest and other noncurrent liabilities, including deferred taxes and provisions, divided by average annual total assets.

^(g)^ Stoxx Europe 600 annual return, expresses the theoretical growth in value of a share holding over a specified period, assuming that dividends are re-invested to purchase additional units of the stock.

The accrual anomaly will be present if there is (1) a significant negative relationship between quartiles of accruals and size-adjusted abnormal returns and (2) significant positive size-adjusted hedge returns.

### 4.1 Long-term operating and working capital accruals

**Fig 1:**
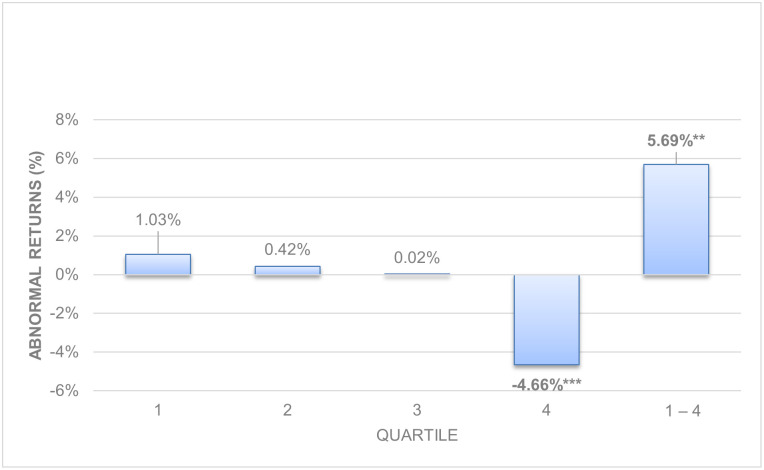
Size-adjusted abnormal returns. Long-term accruals.

**Fig 2:**
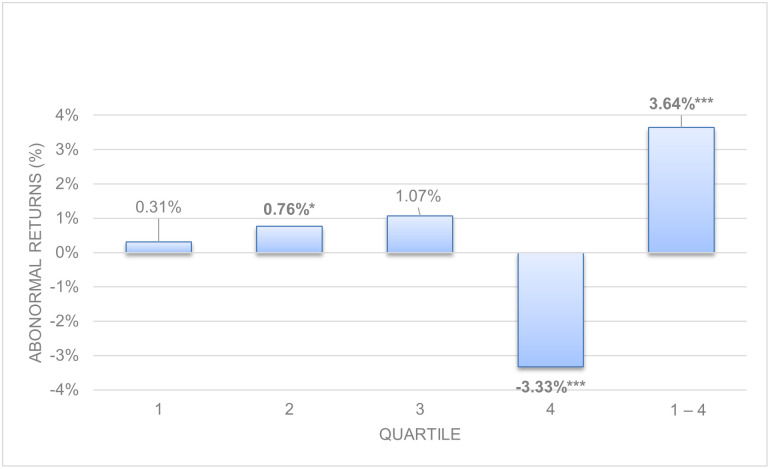
Size-adjusted abnormal returns. Working capital accruals.

Table 2, represented graphically in Figs 1 and 2, shows the abnormal and hedge returns with their p-value for long-term (Panel A) and working capital accruals (Panel B). Long-term accruals show stronger evidence of anomalies than working capital accruals as we theorized in Hypothesis 1, following previous literature [4, 5, 18].

**Table 2:** Size-adjusted abnormal returns.

PANEL A: LONG TERM OPERATIVE ACCRUALS						
**Quartile**	**Accrual cutoff**	**Size-adjusted abnormal return**	**t**	**p value**	**St. Desv.**	**Number of observations**
1	0.0232	1.03%	1.50	0.150	3.16%	4,549
2	0.0858	0.42%	0.63	0.536	3.05%	2,585
3	0.1846	0.02%	0.03	0.977	3.79%	1,218
**4**		-4.66%***	**-3.06**	**0.006**	**6.98%**	**887**
**1–4**		5.69%**	**2.75**	**0.012**	**9.49%**	
PANEL B: WORKING CAPITAL ACCRUALS						
**Quartile**	**Accrual cutoff**	**Size-adjusted abnormal return**	**t**	**p value**	**St. Desv.**	**Number of observations**
1	-0.0213	0.31%	0.44	0.662	3.22%	2,051
**2**	**0.0076**	0.76%*	**1.80**	**0.088**	**1.95%**	**3,576**
3	0.0311	1.07%	1.40	0.177	3.49%	2,075
**4**		-3.33%***	**-4.51**	**0.000**	**3.38%**	**1,537**
**1–4**		3.64%***	**3.47**	**0.002**	**4.80%**	

Significant at *10%, **5%, ***1% levels. Returns are the time-series mean annual buy-and-hold size-adjusted returns [40] beginning in May 2nd. The p-value are based on the standard deviation of a conventional two-tailed t-statistic computed over the 21 annual mean returns in a quartile [2]. Since the break points between quartiles are based on the first year’s cutoffs (1999), the number of observations in each quartile changes. Long term operating accruals are calculated as the change in noncurrent operating assets minus the change in noncurrent operating liabilities divided by average annual total assets. Working capital accruals are calculated as the change in non-cash current assets minus the change in current liabilities less short term financial debt, divided by average annual total assets.

The analysis of the results finds evidence of anomalies for non-decomposed long-term operating accruals. Panel A in Table 2 for long-term operating accruals shows negative returns in the fourth quartile (-4.66%, p = 0.006) and a positive hedge return (5.69%, p = 0.012). We also find evidence of working capital accruals anomalies, but lower in absolute value than long term operating accruals. Panel B of Table 2 for working capital accruals shows three significant returns, one positive in the second quartile (0.76%, p = 0.088); one negative in the last quartile (-3.33%, p = 0.000) and a positive hedge return (3.64%, p = 0.002). In both cases, according to the earnings fixation hypothesis, investors obtain a negative return in the high accrual quartile when they ignore the composition of profits, expect higher future profits and are surprised the following year when the lower persistence of accruals pushes profits downward.

### 4.2 Decomposed long-term asset and liability accruals

**Fig 3:**
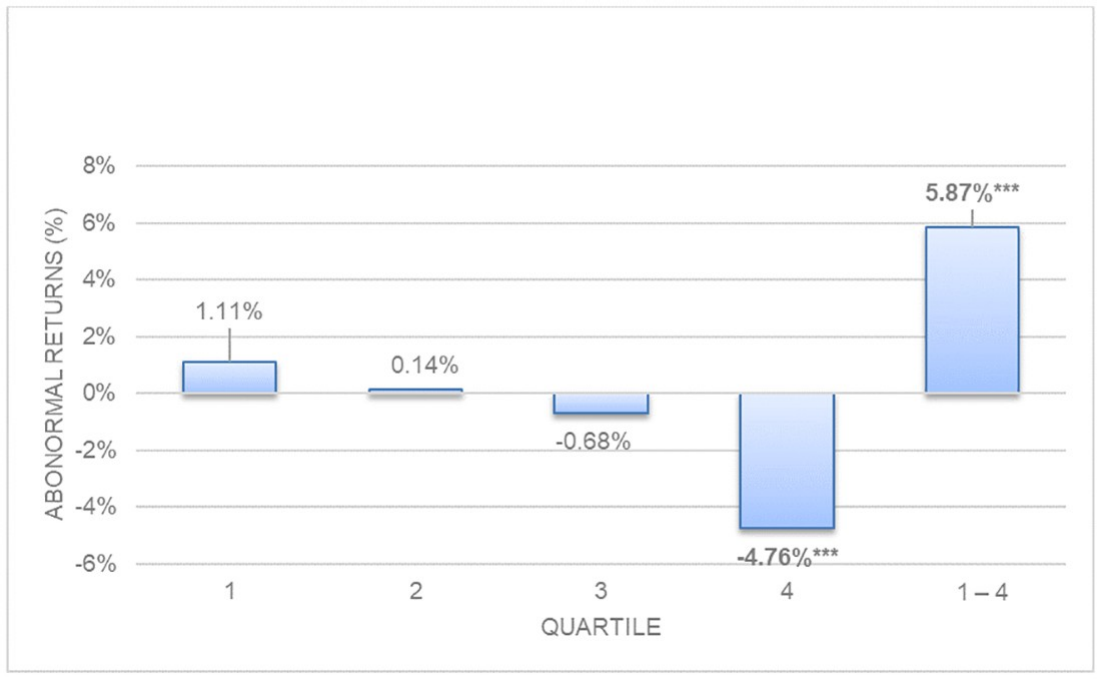
Size-adjusted abnormal returns. Long-term operating asset accruals.

**Fig 4:**
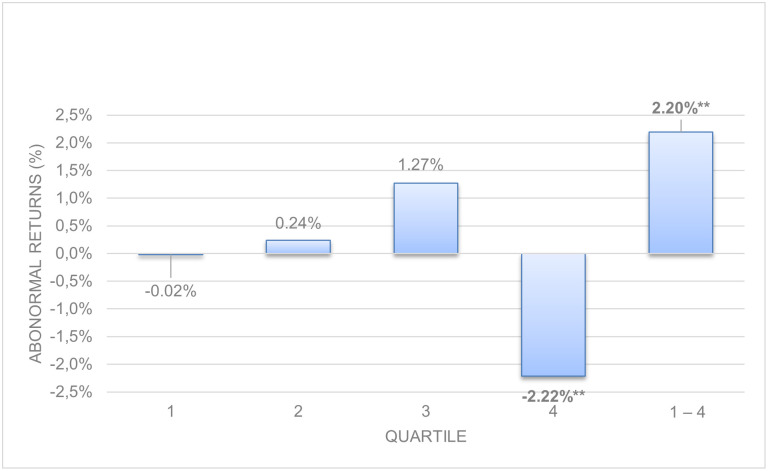
Size-adjusted abnormal returns. Long-term operating liabilities accruals.

Table 3, represented graphically in Figs 3 and 4, shows the abnormal and hedge returns with p-values for decomposed long-term asset and liability accruals. Quartile 1–4 represents hedge returns, obtained deducting from the return in quartile 1 the return in quartile 4.

**Table 3:** Size-adjusted abnormal returns.

PANEL A: NON CURRENT ASSETS ACCRUALS						
**Quartile**	**Accrual cutoff**	**Size-adjusted abnormal return**	**t**	**p value**	**St. Desv.**	**Number of observations**
1	0.0328	1.11%	1.46	0.161	3.51%	4,986
2	0.0901	0.14%	0.14	0.891	4.49%	2,104
3	0.1891	-0.68%	-0.45	0.657	6.95%	1,214
**4**		-4.76%***	**-3.49**	**0.002**	**6.25%**	**935**
**1–4**		5.87%***	**3.10**	**0.006**	**8.67%**	
PANEL B: NON CURRENT LIABILITIES ACCRUALS						
**Quartile**	**Accrual cutoff**	**Size-adjusted abnormal return**	**t**	**p value**	**St. Desv.**	**Number of observations**
1	0.0232	-0.02%	-0.03	0.976	2.81%	3,185
2	0.0858	0.24%	0.39	0.700	2.83%	2,541
3	0.1846	1.27%	1.54	0.139	3.76%	1,744
**4**		-2.22%**	**-2.80**	**0.011**	**3.63%**	**1,769**
**1–4**		2.20%**	**2.30**	**0.032**	**4.38%**	

Significant at *10%, **5%, ***1% levels. Returns are the time-series mean annual buy-and-hold size-adjusted returns [40] beginning in May 2nd. The p-values are based on the standard deviation of a conventional two-tailed t-statistic computed over the 21 annual mean returns in each quartile [2]. Since the break points between quartiles are based on the first year’s cutoffs (1999), the number of observations in each quartile changes. Long term operating asset accruals are calculated as the change in intangible assets, property, plant, equipment and other noncurrent assets divided by average annual total assets. Long term operating liability accruals are calculated as the change in minority interest and other noncurrent liabilities, including deferred taxes and provisions, divided by average annual total assets.

Both long-term operating asset and liability accruals show anomalies, and both present significant hedge returns. However, long-term (noncurrent) operating asset accruals show stronger evidence of anomalies (5.87%, p = 0.006) than long-term operating liabilities accruals (2.20%, p = 0.032) as we theorized in Hypothesis 2. Panels A and B of Table 3, and Figs 3 and 4 show that sized adjusted abnormal returns are lower (-4.76%) for quartile 4 (highest accruals) for long term asset accruals than for long term liabilities (-2,22%): when long-term operating accruals are decomposed into their asset and liability accruals, higher evidence of anomalies appears. This evidence is stronger in long-term operating asset accruals than in long-term operating liabilities accruals, consistent with Chichernea [7] who finds significant relationships of bond returns in the USA market with noncurrent operating assets accruals. These findings suggest that investors are overly optimistic and exaggerate their positive expectations about the following year´s profits derived from higher long-term operating asset accruals, and subsequently are surprised by a negative return when profits are lower than expected.

### 4.3 Robustness check

Table 4 shows the main results of both regression models: the first one with the working capital accruals and long-term accruals as independent variables; and the second one, decomposing the long term by its components: noncurrent operating assets accruals and noncurrent operating liabilities accruals. In all cases, the regression is significant at 1%, and all accruals are significant at 1%, with negative coefficients, except for the long-term operating liabilities accruals.

**Table 4:** Regreions model.

PANEL A: WORKING CAPITAL VS LONG TERM OPERATIVE ACCRUALS				
**Accural**	**Standarized Beta Coefficient**	**t**	**p value**	**St. Error**
TWCACC	-0.043***	-4.17	0.000	0.056
TLTACC	-0.062***	-5.99	0.000	0.019
PANEL B: WORKING CAPITAL VS LONG TERM ASSETS AND LIABILITIES ACCRUALS				
**Accural**	**Standarized Beta Coefficient**	**t**	**p value**	**St. Error**
TWCACC	-0.042***	-3.99	0.000	0.056
TNCOAACC	-0.065***	-5.71	0.000	0.019
TNCOLACC	0.002	0.14	0.886	0.069

Significant at *10%, **5%, ***1% levels. TWCACC is the working capital accrual scaled by total assets; TLTACC is the long term operating accrual scaled by total assets; TNCOAACC is the noncurrent operating assets accruals scaled by total assets; and TNCOLACC is the non-current operating accruals scaled by total assets.

The regression analysis presents similar results to the conventional two-tailed t-statistic seen above. All the coefficients are negative (except the noncurrent liabilities accrual), which means that abnormal returns are negative when higher accruals appear in the previous year. The long-term operating coefficient is more elevated in absolute value than working capital, and the long-term operating assets coefficient is the highest.

### 4.4 Countercyclical behavior

**Fig 5:**
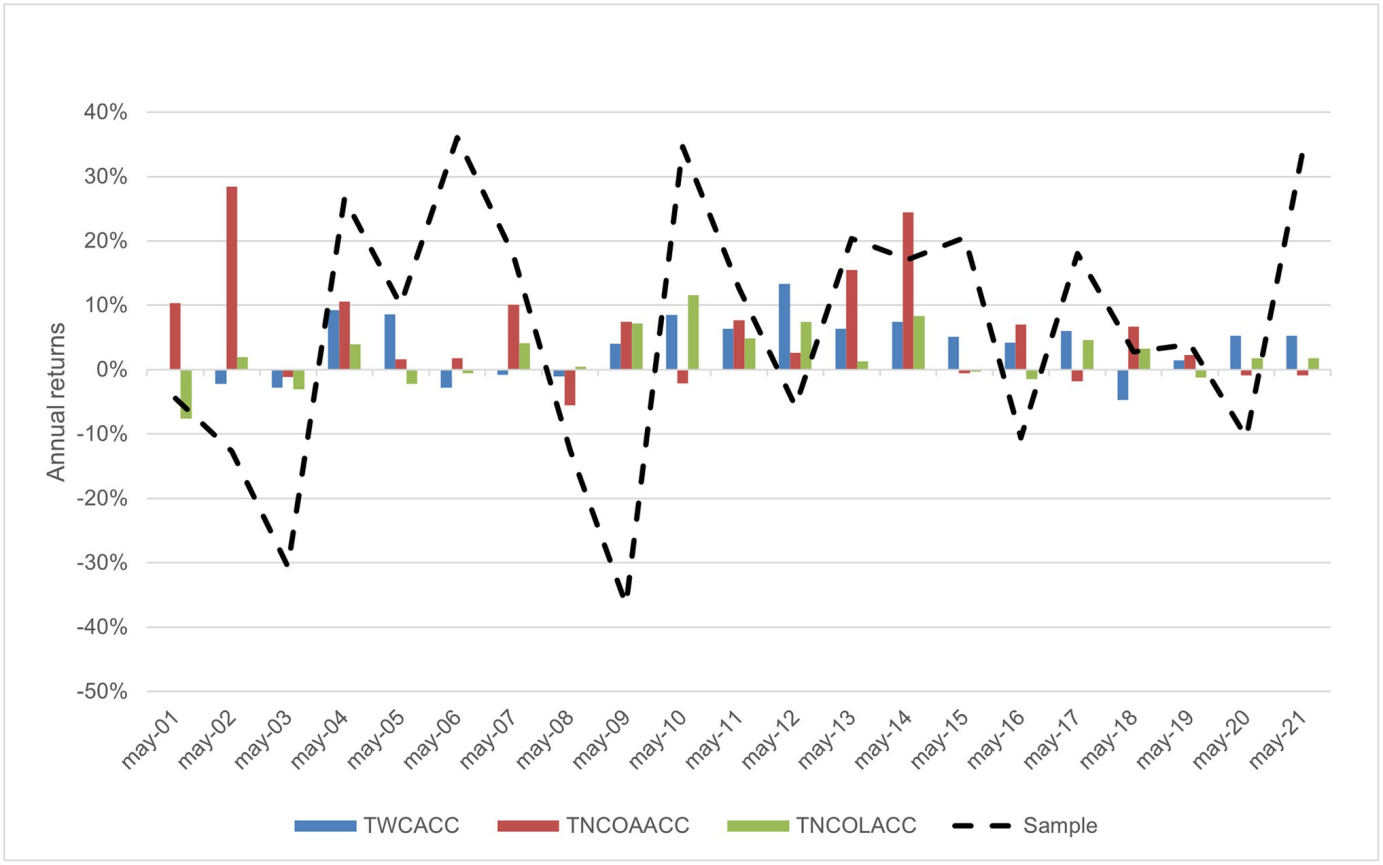
Comparative annual return distribution.

**Fig 6:**
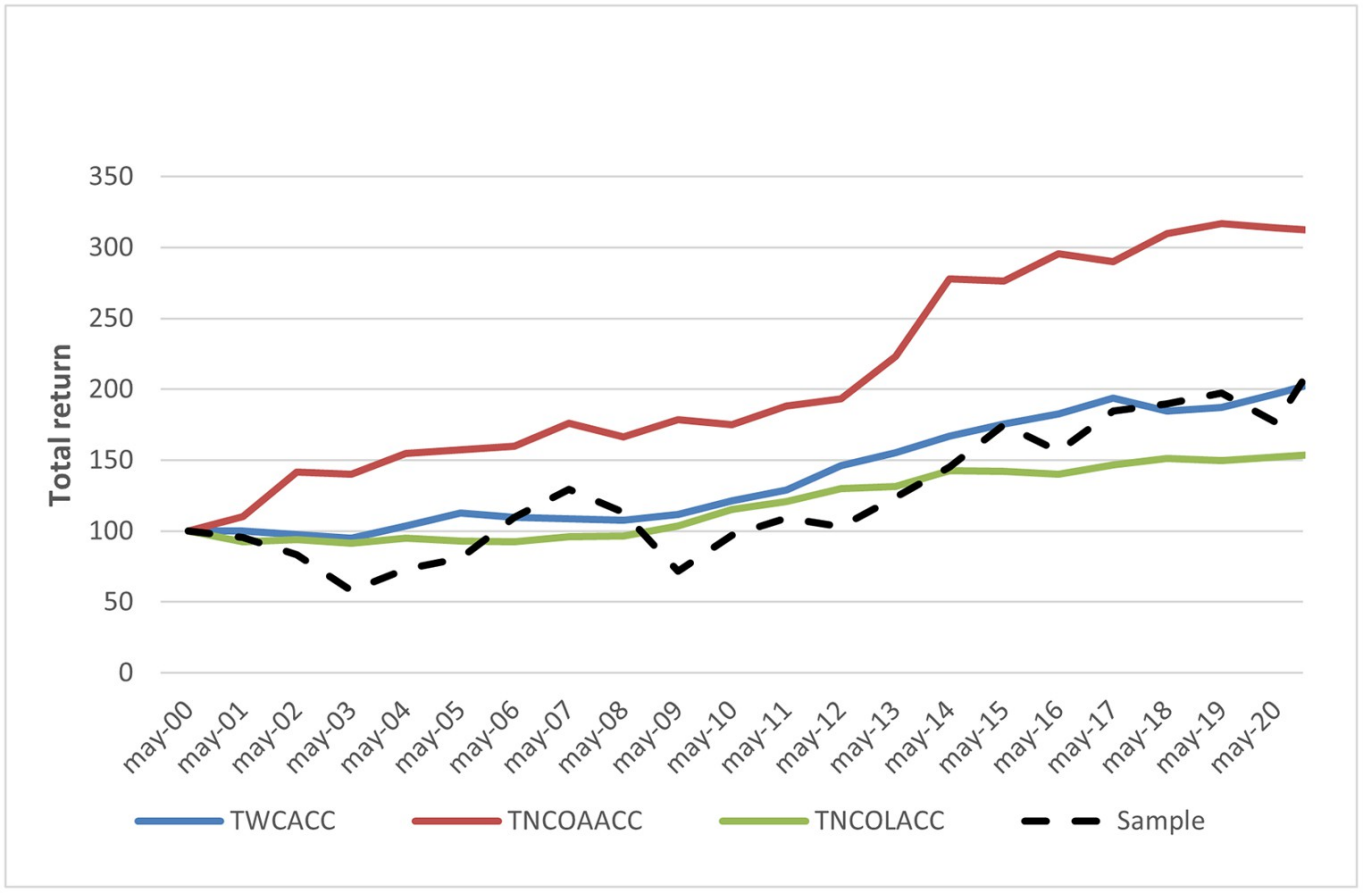
Comparative total returns.

Fig 5 shows the comparative annual return distribution in the period of the portfolio stock returns for the sample trading strategy (Stoxx Europe 600 index return), for the working capital accrual, the long-term operating liabilities accrual, and the long-term operating asset accrual-based strategies. Fig 6 shows the total return for the sample trading strategy, the working capital, the long-term operating asset, and the long-term operating liabilities accrual-based strategies. The compounded annual growth rates for the period are 6.25%, 3.64% 5.87%, and 2.20%, respectively for each trading strategy (see Tables 1–3). However, although sample annual average growth is higher than the other strategies, there is a crucial difference between them when the volatility of the annual returns is observed. Annual returns for both the long-term asset and the working capital accrual trading strategies are clearly less volatile (with a standard deviation of 8.67% and 4.80%, respectively) than those of the sample strategy (standard deviation of 20.56%).

### 4.5 Discussion

Our hypotheses testing delivers the following results:

Our evidence on our first hypothesis H1, that the long-term operating accrual anomaly is higher than the working capital accrual anomaly, is supportive when long-term operating accruals are not decomposed into their asset and liability constituents, and is also consistent with it when long-term operating accruals are decomposed: long-term operating asset accruals show a stronger negative relationship to stock returns and a higher hedge return than working capital accruals. Long-term operating liability accruals, produce a significant hedge return, but lower than the asset component of the long-term operating accruals, confirming our second hypothesis (H2), that the decomposed long-term operating asset accrual anomaly is higher than the long-term operating liability accrual anomaly. These results are consistent with previous studies [1, 18] discussed in the literature reviewed and present the same conclusions when a conventional t-statistics or a regression model are applied.

Our third hypothesis H3, that an accrual-based trading strategy is countercyclical, finds supporting evidence for working capital and long-term operating accruals, especially for its asset components. This finding is consistent with Chichernea *et al*. [7], who observe a stock return dispersion increase in recession periods and a higher correlation of return dispersion with hedge returns for noncurrent than for working capital accruals in the USA. It is also consistent with Wu *et al*. [44], who observe that expected returns to accruals-based trading strategies are time-varying and countercyclical, given that firms optimally adjust their accruals in response to discount rate changes and lower discount rates associated with recession periods typically increase accruals. The observed protection of portfolio values could make the long-term asset accrual trading strategy of great interest to practitioners.

## 5. Conclusion

The main contribution of this paper compared to previous studies on the accrual anomaly is the finding of an implementable long-term operating asset accrual trading strategy that produces steady stock returns and greatly reduces the volatility of annual stock returns versus a sample trading strategy. Such a strategy could be of great interest to practitioners. This paper uses a new approach to the accrual anomaly by focusing on long-term operating accruals, rather than short-term accruals, as an accounting measure to identify abnormal returns. This change in reference has the potential to more accurately identify and predict higher abnormal returns, as extensive literature assigns lower reliability to long-term accruals than to current accruals and relates lower reliability with major anomalies. The vast majority of existing studies on the anomaly focus on short-term or total accruals, with few specific studies on long-term accruals to date.

Following this reasoning, the main finding of this research on a stock portfolio extracted from the Stoxx Europe 600 index in the period 1999–2019 is that undecomposed long-term operating accruals are better predictors of abnormal returns than working capital accruals. When decomposed, long-term operating asset accruals are even better able to identify higher anomalies than working capital accruals which, in turn, help identify higher anomalies than long-term operating liability accruals. This could be the result of higher investor sensitivity, when estimating future earnings, to changes in long-term investments than to changes in working capital, and in turn, to changes in long-term operating liabilities. When the anticipated higher earnings from long-term investments are not materialized, or do not meet expectations, investor´s disappointment hurts stock returns harder than when the failed anticipated higher earnings come from increases in working capital or from decreases in long-term liabilities.

Our computed working capital accruals show substantially lower positive hedge returns than those observed in many US studies. A possible explanation could be that the accrual anomaly is most likely to occur in common rather than in civil law countries, probably as a consequence of different degrees of implementation of the fair value criteria. Despite the fact that the Stoxx Europe 600 index contains more stocks than many European national indexes on which anomalies have been studied, some limitations of this study may come from the need to divide the sample into quartiles instead of the deciles used in many USA investigations, as the size of European stock markets is substantially smaller than that of the NYSE and NASDAQ. This may reduce the accuracy of the results, although quartiles allow sufficiently large subportfolios to calculate significance. On the other hand, the sample suffers from some degree of heterogeneity because the Stoxx Europe 600 index corresponds to nineteen European countries with different accounting rules where the accrual anomaly can occur to different degrees, being highest in the United Kingdom according to numerous studies. An analysis of individual countries could yield different results from those presented in this article and shed new light on the role that the degree of implementation of the fair value criteria may actually play in the accrual anomaly. A comparative analysis of US companies with EUROPE companies becames challenging due to the different info contented in FASB and IASB standards, and the comparison among different sectors.

## Supporting information

S1 File (ZIP)

S2 File (ZIP)

## References

[1] Detzel A, Schaberl P, Strauss J. There are two very different accruals anomalies. Eur Fin Manag [Internet]. 2018;24(4):581–609. Available from: 10.1111/eufm.12162

[2] Sloan R. (1996). Do stock prices fully reflect information in accruals and cash flows about future earnings? *The Accounting Review* 71, 289–316. https://www.jstor.org/stable/248290

[3] Larson C., Sloan R., & Zha Giedt J. (2018). Defining, Measuring and Modeling Accruals: A Guide for Researchers. *Review of Accounting Studies* 23 (3): 827–871. https://ssrn.com/abstract=2952601 doi: 10.2139/ssrn.2952601

[4] Richardson S.; Sloan R.; Soliman M. & Tuna I. (2005). Accrual reliability, earnings persistence and stock prices. *Journal of Accounting and Economics* (September) 437–85. doi: 10.1016/j.jacceco.2005.04.005

[5] Downes J. F., Kang T., Kim S., & Lee C. (2019). Does the Mandatory Adoption of IFRS Improve the Association between Accruals and Cash Flows? Evidence from Accounting Estimates. *Accounting Horizons*, 33(1), 39–59. doi: 10.2308/acch-52262

[6] Zhu W. Accruals and price crashes. Rev Account Stud 21, 349–399 (2016). doi: 10.1007/s11142-016-9355-1

[7] Chichernea D., Holder A., & Petkevich A. (2019). Decomposing the accrual premium: The evidence from two markets. *Journal of Business Finance and Accounting*, 46(7–8), 879–912. doi: 10.1111/jbfa.12394

[8] Iñiguez S., R. & Poveda F., F. (2008). Persistence in accounting profit and its components: implications from the measurement of accruals. *Spanish Journal of Finance and Accounting /* *Revista Española de Financiación y Contabilidad*, 37 (137), 33–61. doi: 10.1080/02102412.2008.10779638

[9] Gonçalves T, Gaio C, & Lélis C. (2019). Accrual Mispricing: Evidence from European Sovereign Debt Crisis. *Research in International Business and Finance*, doi: 10.1016/j.ribaf.2019.101111

[10] Zhang X. F. (2007). Accruals, investment, and the accrual anomaly. *The Accounting Review* 82, 1333–1363. doi: 10.2308/accr.2007.82.5.1333

[11] Jones J. J. (1991). Earnings Management During Import Relief Investigations. *Journal of Accounting Research* 29 (2), 193–228. doi: 10.2307/2491047 https://www.jstor.org/stable/2491047

[12] Teoh S., & Wong T. (2002). Why New Issues and High-Accrual Firms Underperform: The Role of Analysts’ Credulity. *The Review of Financial Studies*, 15(3), 869–900. February 25, 2021, http://www.jstor.org/stable/2696724

[13] Keim D. B. (2008). Financial Market Anomalies. In The New Palgrave Dictionary of Economics. 2nd Ed., Eds. Steven N. Blume Durlauf y Lawrence E. Gordonsville: Palgrave Macmillan.

[14] Shi L. & Zhang H. (2012). Can the earnings fixation hypothesis explain the accrual anomaly? *Review of Accounting Studies* 17, 1–21. doi: 10.1007/s11142-011-9171-6

[15] Jensen M. C. (2005). Agency Costs of Overvalued Equity. *Financial Management* 34 (1), 5–19. 10.1111/j.1755-053X.2005.tb00090.x

[16] Kothari, S. P.; Loutskina, E. & Nikolaev, V. V. (2006). Agency Theory of Overvalued Equity as an Explanation for the Accrual Anomaly. *Working Paper*, http://ssrn.com/abstract=871750 or 10.2139/ssrn.871750 [February 1^st^ 2015]

[17] Papanastasopoulos G. A., & Tsiritakis E., (2015). The accrual anomaly in Europe: The role of accounting distortions. *International Review of Financial Analysis*, 41, 176–185. 10.1016/j.irfa.2015.06.006

[18] Papanastasopoulos G.A. (2015). Accruals, growth, accounting distortions and stock returns: The case of FRS3 in the UK. *North American Journal of Economics and Finance*, Vol. 33, pp. 39–54. 10.1016/j.najef.2015.03.003

[19] Aldahray A. & Alnori F. (2021). Impact of regulatory environment on accruals manipulation of bankrupt firms. *Spanish Journal of Finance and Accounting /* *Revista Española de Financiación y Contabilidad*, 50:1, 114–142, doi: 10.1080/02102412.2020.1735209

[20] Li L.; Monroe G.S.; Wang J.J. (2020). State Ownership and Abnormal Accruals in Highly-Valued Firms: Evidence from China. *Journal of Contemporary Accounting & Economics*. 10.1016/j.jcae.2020.100223

[21] Papanastasopoulos G.; Thomakos D. & Wang T. (2013). Corporate financing activities, fundamentals to price ratios and the cross section of stock returns. *Journal of Economic Studies* 40 (4), 493–514. 10.1108/JES-08-2011-0097

[22] Hardouvelis G.; Papanasatasopoulos G.; Thomakos D. & Wang T. (2012). External Financing, Growth and Stock Returns. *European Financial Management* 18, 790–815. 10.1111/j.1468-036X.2012.00656.x

[23] Livnat J. & Santicchia M. (2006). Cash Flows, Accruals, and Future Returns. *Financial Analysts Journal* 2 (4), 48–61. 10.2469/faj.v62.n4.4186

[24] Hafzalla N.; Lundholm R. & Van Winkle M. (2011). Percent accruals. *The Accounting Review* 86, 209–236. 10.2308/accr.00000011

[25] Lev B. & Nissim D. (2006). The persistence of the accruals anomaly. *Contemporary Accounting Research* 23 (1), 1–34. 10.1506/C6WA-Y05N-0038-CXTB

[26] Lafond, R. (2005). Is the accrual anomaly a global anomaly?. *Working Paper*, SSRN eLibrary: http://ssrn.com/abstract=782726 [consulted February 2^nd^ 2015]. 10.2139/ssrn.782726

[27] Pincus M.; Rajgopal S. & Venkatachalam M. (2007). The Accrual Anomaly: International Evidence. *The Accounting Review* 82 (1), 169–203. 10.2308/accr.2007.82.1.169

[28] Leippold M. & Lohre H. (2012). Data snooping and the global accrual anomaly. *Applied Financial Economics*, 22, 509–535. 10.1080/09603107.2011.631892

[29] Doukakis L. & Papanastasopoulos G. (2014). The accrual anomaly in the U.K. stock market: Implications of growth and accounting distortions. *Journal Of International Financial Markets*, *Institutions & Money* 32, 256–277. 10.1016/j.intfin.2014.06.006

[30] Kaserer C. & Klingler C. (2008). The accrual anomaly under different accounting standards—Lessons learned from the German experiment. *Journal of Business Finance & Accounting* 35, 837–859. doi: 10.1111/j.1468-5957.2008.02089.x

[31] Peek E., Meuwissen R., Moers F., & Vanstraelen A. (2013). Comparing Abnormal Accruals Estimates Across Samples: An International Test. European Accounting Review 22 (3) 533–572. 10.1080/09638180.2012.746518

[32] Sullivan M. & Zhang A. J. (2011). Are investment and financing anomalies two sides of the same coin? *Journal of Empirical Finance*, 18 (4), 616–33. 10.1016/j.jempfin.2011.05.007

[33] Titman S.; Wei K. C. J. & Xie F. X. (2004). Capital investments and stock returns. *Journal of Financial and Quantitative Analysis* 39 (4), 677–700. 10.1017/S0022109000003173

[34] Cooper M. J.; Gulen H. & Schill M. J. (2008). Asset growth and the cross-section of stock returns. *The Journal of Finance* 63, 1609–1651. 10.1111/j.1540-6261.2008.01370.x

[35] Li X.; Becker Y. & Rosenfeld D. (2012). Asset Growth and Future Stock Returns: International Evidence. *Financial Analysts Journal* 68, 51–62. 10.2469/faj.v68.n3.4

[36] Fama E. y French K. (1993). Common Risk Factors in the Returns of Stocks and Bonds. *Journal of Financial Economics* 33, 3–56.

[37] Palmon D.; Sudit E. F. & Ari Yezegel (2008). The Accruals Anomaly and Company Size. *Financial Analysts Journal* 64 (5), 47–60. 10.2469/faj.v64.n5.6

[38] Keim D. B. (1983). Size-Related Anomalies and Stock Return Seasonality. *Journal of Financial Economics* 12, 13–32. 10.1016/0304-405X(83)90025-9

[39] Lyon JD, Barber BM, Tsai C-L. Improved methods for tests of Long-Run abnormal stock returns. J Finance [Internet]. 1999;54(1):165–201. Available from: 10.1111/0022-1082.00101

[40] Jones DA, Smith KJ. Comparing the value relevance, predictive value, and persistence of other comprehensive income and special items. account rev [Internet]. 2011;86(6):2047–73. Available from: 10.2308/accr-10133

[41] Kraft A, Leone AJ, Wasley C. An Analysis of the Theories and Explanations Offered for the Mispricing of Accruals and Accrual Components: Mispricing of accruals. J Acc Res [Internet]. 2006;44(2):297–339. Available from: 10.1111/j.1475-679x.2006.00202.x

[42] Wei KCJ, Xie F. Accruals, capital investments, and stock returns. Fin Anal J [Internet]. 2008;64(5):34–44. Available from: 10.2469/faj.v64.n5.5

[43] Alford AW, Jones JJ, Zmijewski ME. Extensions and violations of the statutory SEC form 10-K filing requirements. J Account Econ [Internet]. 1994;17(1–2):229–54. Available from: 10.1016/0165-4101(94)90011-6

[44] Wu J (ginger), Zhang LU, Zhang XF. The q‐theory approach to understanding the accrual anomaly. J Acc Res [Internet]. 2010;48(1):177–223. Available from: 10.1111/j.1475-679x.2009.00353.x.

